# Influencing factors of physicians’ prescription behavior in selecting essential medicines: a cross-sectional survey in Chinese county hospitals

**DOI:** 10.1186/s12913-019-4831-5

**Published:** 2019-12-19

**Authors:** Xin Zhou, Xueting Zhang, Linsheng Yang, Xu Hu, Aizong Shen, Xiaohui Huang, Xuefeng Xie

**Affiliations:** 1 Department of Basic and Clinical Pharmacology, Anhui Institute of Innovative Drugs, School of Pharmacy, Anhui Medical University, 81 Meishan Road, Hefei, 230032 Anhui People’s Republic of China; 2 Department of Epidemiology and Health Statistics, School of Public Health, Anhui Medical University, Hefei, 230032 Anhui China; 3 Department of Pharmacy, Anhui Provincial Hospital, Hefei, 230001 Anhui China

**Keywords:** Health care system reform, Essential medicine, Prescription behavior, Rational administration

## Abstract

**Background:**

To explore the key factors affecting prescription practices of essential medicines in Chinese county hospital. National essential medicine policy (NEMP) plays important roles in health care system, especially in developing countries. As a fundamental component in the Chinese health system reform, NEMP was implemented in primary health care institutions during the first stage of reform. As it is rolled out, priority usage and zero-mark-up policy of essential medicines are to be applied in every government-run healthcare institution. However, the intention and influence factors of physicians on priority selecting essential medicine remains unclear.

**Methods:**

Based on the theory of planned behavior, a cross-sectional questionnaire survey was conducted to analyze physicians’ intention, attitude, subjective norms (SNs) and perceived behavioral control (PBC) on prescrictions and their actual behavior on selection of essential medicines.

**Results:**

Two hundred eighty-two physicians participated in the structural questionnaire interview. The final structural equation model reflected influencing factors affecting physicians’ prescribing behavior (*χ*^2^/df = 1.32, GFI = 0.99, IFI = 0.99). Structural equation model analysis showed that attitude, other influencers and institutional environment, and PBC significantly affected behavioral intention. However, the control extent of cognition behavior of physicians prescribing had no significant positive effect on the priority usage of essential medicines.

**Conclusion:**

Investigation results demonstrate physicians are unaware of NEMP design and implementation plans. To help enhance rational use of essential medicines we suggest educating physicians on the value of NEMP, and integrating the drug shortage report into the essential medicine (EM) bidding system seamless communication with pharmaceutical manufacturers’ credit information system.

## Background

Public health care reform in any country is a massive challenge complicated by the need to maintain high medical quality and optimize resource allocation. China medical and pharmaceutical system reform, referred to as “the new health care reform”, was launched in April 2009 aiming to create universal health coverage and promote quality of medical services [1]. China’s public health institutions are managed in a hierarchical model composed of primary, secondary and tertiary hospitals; this hierarchization is also reflected in the whole process of health care reform. As the most important part of this reform, China’s National Essential Medicines Policy (NEMP) was established in primary hospitals after the first stage of reform. To expanding the national essential medicines coverage, the zero-make-up policy has being carried out. It’s a national reform by sale drugs at ex-factory price, which the profits of medical institutions should be borne by the government to reduce the cost for patients [2]. After implementing the zero-mark-up policy in the essential medicine system, the average cost per clinical visit and drug cost per prescription has decreased [3]. All government-run primary healthcare institutions were directed to prescribe only essential medicines, sold under the zero-mark-up policy based on cost from procurement to retailer [4] . Further, secondary (county-level) and tertiary (upper-level) hospitals are required to prescribe essential medicines preferentially and encouraged to follow the zero-mark-up policy [5]. County public hospitals are the main medical institutions that provide health service for urban and rural populations. They have become important links from the reform of primary medical institutions to the overall implementation in China. Have there been any significant changes of the physicians’ prescription behaviors in selecting essential medicine in county hospitals? If so, has this change met the expectations of policy design? These are all issues that should be of concern.

Anhui Province was one of the earliest provinces to implement these reforms, establishing graded diagnosis and treatment systems, zero-mark-up policy for medicines, and a “two-envelop” drug bidding procurement system to separate bidding from and commercial activities [6, 7]. Anhui health care reform model is very specific and understandable for China’s health system which has represented pilot policies later adopted throughout China. The previous study analyzed the use of essential medicines in county public hospitals during years 2011 to 2013 before and after the national health care reform in Anhui Province. Surprisingly, results show essential medicine consumption in primary health care institutions was less than expected [8]. This indicates county public hospitals may be neglecting proper clinical use of essential medicines.

At present, influencing factors of essential medicine usage in county public hospitals have not been studied. Based on results of these pre-surveys our current study used county public hospitals of Anhui Province as the study sample and explored why insufficient attention has been paid on essential medicines in upper-level hospitals. Hope to provide decision-making guidelines and methodological structure to ensure rational use of essential medicines in China health care system.

## Methods

### Study design

Anhui Province is located in eastern middle China and includes 17 cities with a population of 61.4 million. Stratified randomly sampling was used to ensure equal representation according to the economic and regional distribution of Anhui Province. Two county hospitals from a city in northern, southern and central regions were stratified randomly sampled for a total of six target hospitals. A cross-sectional questionnaire survey was conducted from September to November 2015 based on drug utilization from years 2011 to 2013 before and after the national comprehensive reform [9]. With the assistance of the hospital management department, physicians with prescribing right in six sample hospitals were all invited to participate this anonymous survey by the paper-base questionnaire, during an internal meeting (all physicians are required to attend), and the questionnaires were taken back on the spot. Eventually, 302 physicians volunteered to answer this survey and showed positively to some extent.

### Research model and hypotheses

**Fig. 1:**
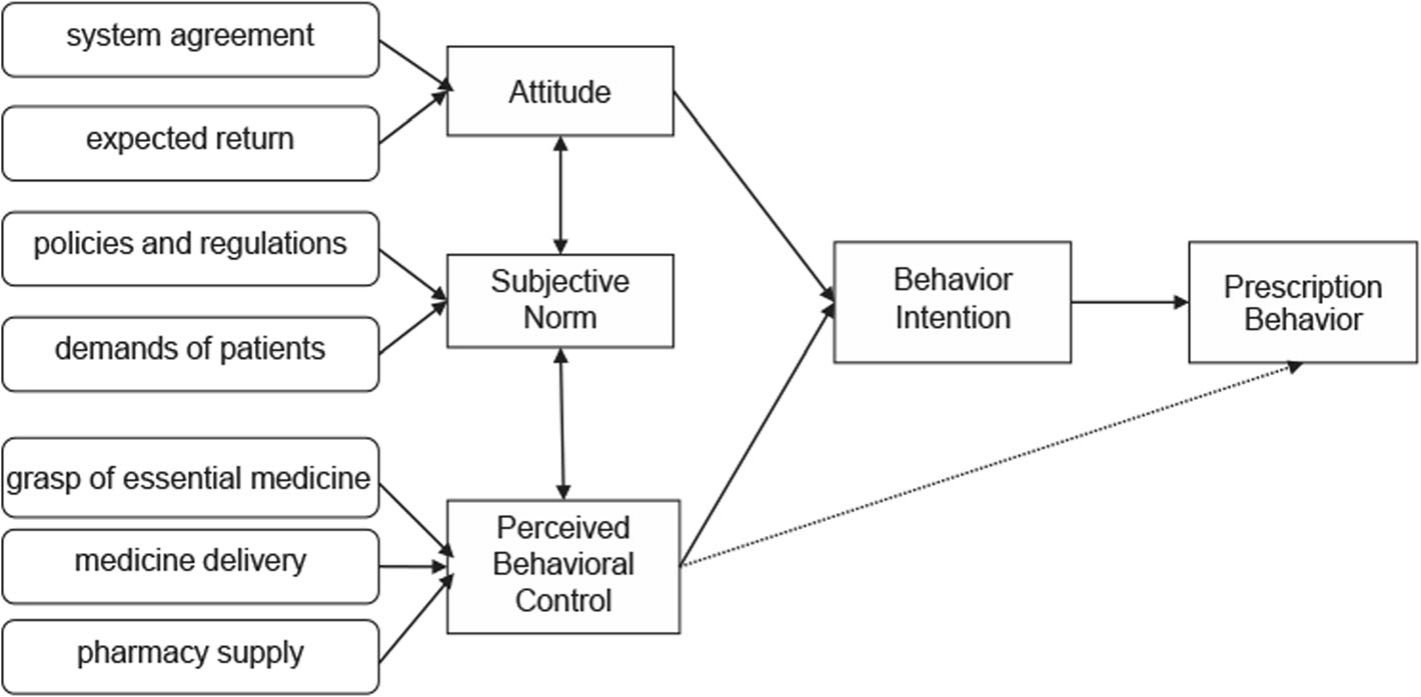
Research model of the key factors influencing the priority usage of essential medicines

Physician prescription behavior drives use of essential medicines in public hospitals. This study qualitatively and quantitatively analyzed the decision-making approach of physicians. The theory of planned behavior (TPB) is a theory that explained the general decision-making process of individuals’ behavior from the perspective of information processing and the value of expectation [10], which including: attitude towards the behavior (AB), subjective norms (SNs), perceived behavioral control (PBC), behavioral intention (BI) and actual behavior. The TPB believes that actual behavior is determined by the individual’s BI; behavioral intentions are determined by AB, SNs, and PBC. Based on the TPB [11, 12], we developed a research model to investigate factors influencing behavioral intention (Fig. 1). Five hypotheses are listed below:

- H1: Physicians’ understanding of essential medicine system has a positive effect on prescription behavior;
- H2: Institutional environment has a positive effect on the behavior intention of basic prescriptions;
- H3: The control extent of cognition behavior has a positive impact on the behavior intention of the basic prescription medicines;
- H4: The control extent of cognition behavior has a positive effect on actual behavior of the priority of essential medicines;
- H5: The behavior intention of the priority usage of essential medicines has a positive effect on the actual behavior of essential medicine prescriptions. (Fig. 1)

### Questionnaire design

A cross-sectional survey was conducted to test the hypotheses. The data on various constructs of the research models were collected using the structured questionnaire. Questionnaire items were designed on the basis of previous qualitative interview and literature (Additional file 1) [13–15]. Forty physicians were enrolled in the pilot investigation based on the questionnaire draft; reliability and validity of the questionnaire were analyzed. After reasonable adjustment according to the pilot investigation, a formal questionnaire for this study was designed that consisted of three parts: (1) cover letter explaining the contents of NEMP and the purpose of the study; (2) items related to the respondents’ demographics and relevant characteristics, including gender, education level, years of practice in hospital and current position; (3) 26 items were generated on the basis of five main constructs of the TPB including the attitude of physicians’ behavior towards priority prescription of essential medicines (four items), SNs (six items), physicians’ PBC (five items), behavioral intention (BI) of EM-first prescription (five items) and prescribing behavior (six items). All items were scored on a seven-point Likert Scale. To ensure consistency and accuracy, interviews were conducted in six county public hospitals.

### Data analysis

The demographic data were summarized as frequencies and percentages, confirmatory factor analysis (CFA) was performed using structural equation model (SEM) to investigate the relationships among factors. Data were analyzed using AMOS 17.0 (Amos Development Corporation). The associations among AB, SNs, PBC, BI and physicians’ behavior towards priority prescription of essential medicines were examined by calculating the Pearson correlations coefficient. According to recommendations by Hoyle and Panter [16], we reported multiple indexes of fit including chi-square, goodness-of-fit index (GFI), incremental-fit index (IFI), comparative-fit index (CFI), and root mean square error of approximation (RMSEA). We used the bootstrap procedure to test indirect effects and reported the bias-corrected 95% confidence intervals (CIs) for the size of each indirect effect [17] .

## Results

### Demographics of respondents

One’s attitude impacts a preference to perform or not perform a behavior. This study was designed to validate the evaluation system of physician behavior towards priority prescription of essential medicines. Of distributed surveys, 302 were returned with responses. The questionnaire was judged to be invalid if more than 15% items were missed or all the answers were selected as the same option and so on. From the 302, 282 were usable for data analysis, for an overall response rate of 80.57%. According to the description of the sample size of structural equation [18, 19], each factor should have at least three observable indicators, and the total sample size should not be less than 100. Therefore, all 282 samples met the requirement of sample size of SEM.

The interviewees who were the physician on duty were invited to complete the structured questionnaire. They were accompanied by administrative hospital staff, two well-trained research assistants (RAs). Among the respondents, 201 (71.28%) were male, 81.21% had Bachelor’s degree, 47.87% were junior physicians, 42.55% were the physician-in-charge, with an average years of practice of 11.07 ± 8.64 (Table 1).

**Table 1:** Demographics and relevant characteristics of participants (*n* = 282)

Variables	Mean	Standard deviation
Age	34.92	7.79
Years of practice	11.07	8.64
	Number	Percent
Gender		
male	201	71.28
female	82	28.72
Education level		
Associate degree and below	40	14.19
Bachelor’s degree	229	81.21
Master’s/Doctoral degree	13	4.60
Current position		
Junior physician	135	47.87
Physician-in-charge	120	42.55
Deputy chief/ chief physician	27	9.58
Work role/department		
Internal Medicine	82	29.08
Surgery	79	28.01
Obstetrics and Gynecology	35	12.41
Pediatrics	26	9.22
Others	60	21.28

Others include Ophthalmology, Otorhinolaryngology and ICU etc

### Prescription influencing factors

We conducted confirmatory factor analysis to investigate influencing factors on physicians’ behavior towards priority prescription of essential medicines in county public hospitals and compared indicators with the originally hypothesized scales. We evaluated model fit using the goodness-of-fit index (GFI), incremental-fit index (IFI), comparative-fit index (CFI), and root mean square error of approximation (RMSEA) (Table 2).

**Table 2:** Goodness of fit of the models

	RMR	GFI	AGFI	PGFI	RMSEA	*χ*^2^	df	*P*
Attitude towards behavior	0.018	0.999	0.989	0.100	0.000	0.617	1	0.432
Subjective Norms	0.070	0.984	0.945	0.281	0.069	13.973	6	0.030
Perceived behavioral control	0.032	0.993	0.978	0.331	0.019	5.513	5	0.357
Behavior Intention	0.020	0.997	0.981	0.133	0.000	1.813	2	0.404
Behavior	0.063	0.990	0.970	0.330	0.029	8.667	7	0.277

*RMR* Root Mean square Residual, *GFI* Goodness-of-fit index, *AGFI* Adjusted Goodness of Fit Index, *PGFI* Parsimony Goodness of Fit Index, *RMSEA* Root mean square error of approximation

Based on the TPB, the theoretical model of key factors influencing the priority usage of essential medicines consisted of five structural dimensions including Attitude towards physicians’ behavior, Subjective Norms, Perceived behavioral control, Behavior Intention and the actual behavior. After analysis of reliability and validity of the questionnaire, we held the second-round argumentation to readjust the survey statements, four observable indicators were eliminated according to the standardized coefficients (< 0.3). Finally, 26 observable indicators were included in the formal questionnaire, and shown the statistical results of 26 observation indicators (Table 3). Such as PBC5, “provision of EM in hospital pharmacy” indicated whether the supply of EM meet the clinical needs of disease treatment. Confirmatory factor analysis showed data of the sample fit the model well.

**Table 3:** Survey statements and categorical confirmatory factor analysis (*n* = 282)

Latent variable	Observable indicators	Standardized coefficient	Standard error	Cronbach’s α
Attitude towards behavior (AB)	AB1 Awareness of the EM system	0.60	0.36	0.755
	AB2 Satisfaction of the EM system	0.89	0.49	
	AB3 Satisfaction of the job statue	0.62	0.38	
	AB4 Change of your income	0.46	0.21	
Subjective Norms (SNs)	SN1 Hospitals give publicity to the EM system	0.83	0.39	0.807
	SN2 Application of hospital formulary	0.87	0.45	
	SN3 Incentive measures made by hospitals	0.49	0.24	
	SN4 Effects on the prescription review system	0.62	0.38	
	SN5 Patients would like to make decisions on choosing medicines	0.39	0.15	
	SN6 Frequency of talking about EM to patients	0.52	0.27	
Perceived behavioral control (PBC)	PBC1 Physician’s knowledge and skills to the EML	0.72	0.42	0.774
	PBC2 Access to EM information	0.82	0.38	
	PBC3 Awareness of the EM price	0.79	0.33	
	PBC4 Recognition of the quality of EM	0.73	0.44	
	PBC5 Provision of EM in hospital pharmacy	0.54	0.29	
Behavior Intention (BI)	BI1 Intention to participate in training	0.71	0.50	0.882
	BI2 Intention to active learning about EM	0.88	0.37	
	BI3 Intention to prescribe EM preferentially	0.87	0.36	
	BI4 Necessary support for implementing EM system in hospital	0.77	0.29	
	BI5 Necessary support for implementing zero-markup policy of medicines	0.62	0.38	
Behavior (B)	B1 Frequency of participating in training	0.51	0.26	0.735
	B2 Enthusiasm of learning about EM	0.61	0.37	
	B3 Recommend EM to patients	0.84	0.30	
	B4 Actual behavior of prescribing EM preferentially	0.63	0.20	
	B5 Implementation of performance evaluation in hospitals	0.32	0.10	
	B6 Weight on the DUR in performance evaluation system	0.35	0.12	

*EM* essential medicine, *EML* essential medicine list, *DUR* drug utilization review, *AB* Attitude towards behavior, *SNs* Subjective Norms, *PBC* Perceived behavioral control, *BI* Behavior Intention, *B* Behavior

### Structural equation model

**Fig. 2:**
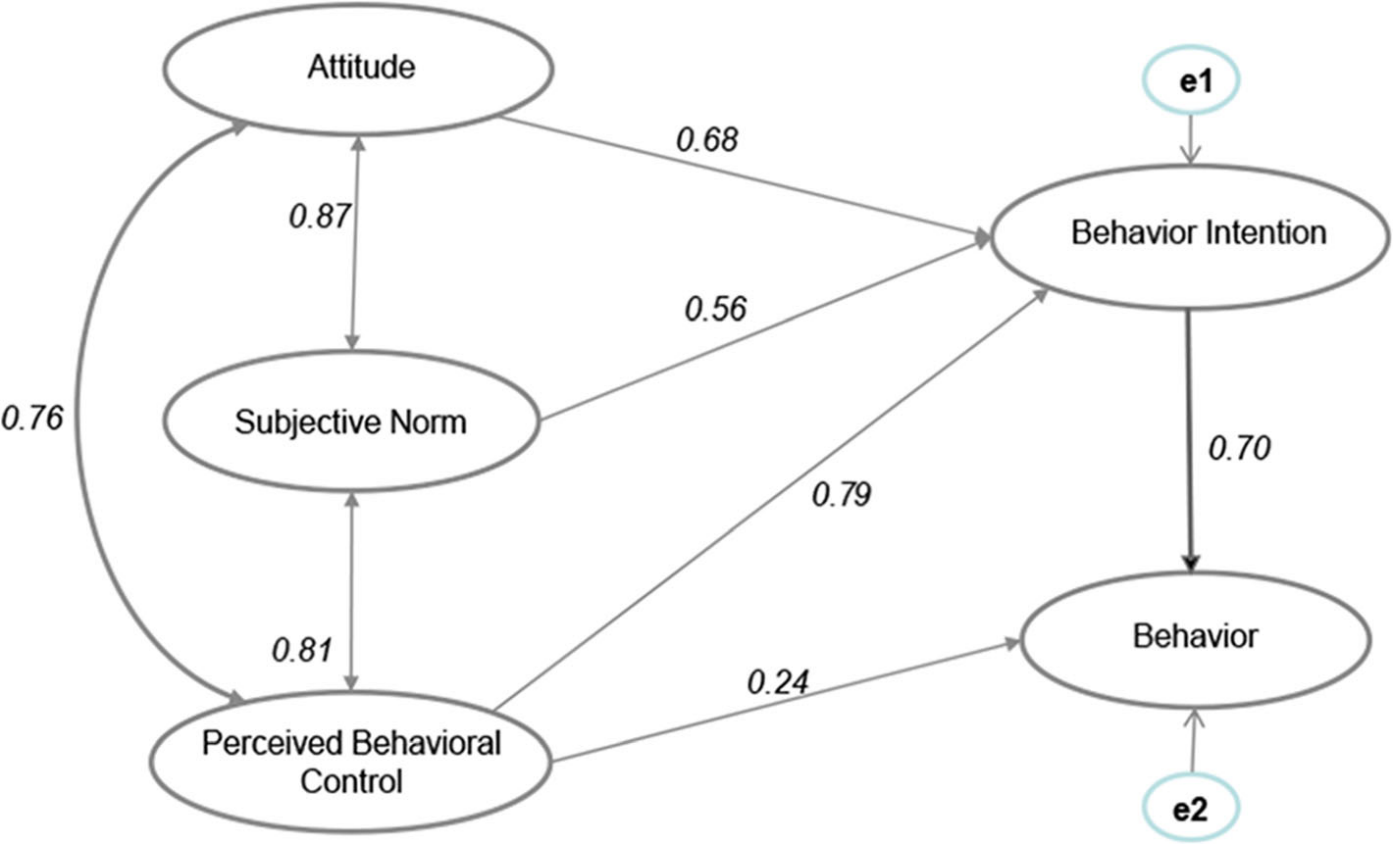
Structural equation model of key factors of priority in the prescription of essential medicines

We used a structural equation model to test the hypothesized correlations among relevant variables (Fig. 1). The standardized parameter estimating the final structural model is shown in Fig. 2. Thus, based on the parsimony principle, the hypothesized model was accepted. The results showed that models fit the data well (*χ*^2^/df = 1.32, GFI = 0.99, IFI = 0.99) (Fig. 2).

Overall, all of the hypotheses were supported by collected information (except H4; t value 1.37). The coefficients indicated that the attitude of physicians on prescribing essential medicine had positive effects on behavioral intention (Fig. 2 and Table 4). Other influencers and institutional environment, SNs, and PBC on the behavior of physicians prescribing essential medicines had a significant positive effect. However, the control extent of cognition behavior of prescription didn’t have a significant positive effect on actual behavior of prescribing essential medicines. Collectively, the behavior intention of physicians may have a direct positive effect on the actual behavior of physicians (Table 4).

**Table 4:** Path coefficients of physicians prescribing essential medicine

Path	Path coefficient	t value	Hypothesis	Result
Attitude → intention to prescribe EM	0.68	5.36*	H1	Supported
Subjective norms → intention to prescribe EM	0.56	2.19*	H2	Supported
Perceived behavior control → intention to prescribe EM	0.79	7.16*	H3	Supported
Perceived behavior control →Behavior	0.24	1.37	H4	Not Supported
intention to prescribe EM → Behavior	0.70	6.84*	H5	Supported

*EM* essential medicine

*P<0.01

## Discussion

The WHO recommends an essential medicine system for developing countries to meet the requirements of basic medical security, and to provide indicators for promoting the quality and safety of clinical drug use. In this regard, China’s national essential medicine system was established as the most important component of healthcare reform since 2009. After implementation at primary health institutions, the NEMP was expanded to county hospitals beginning in 2012, they became key institutions to test reform consequences [20]. Many previous studies focused on the effects of NEMP at primary hospitals [21–23], but few have reported effects at secondary and tertiary hospitals that treat the majority of patients in China and account for a large proportion of drug consumption. In order to consistently serve patients while providing high-quality pharmaceutical service, secondary and tertiary hospitals need encouragement to use essential medicines.

An important issue is whether essential medicines are being prioritized as the new reform policy declares in senior hospitals. Our previous research showed that physicians in county hospitals were under prescribing essential medicines [24]. Given that Anhui Province is representative of China’s health care reform, we used it to analyze the key factors influencing the priority usage of essential medicines.

We used TPB to create the theoretical framework to explore the correlations among the key variables, including attitude, PBC, SNs and physicians’ intention. We found the TPB model was suitable for explaining the physicians’ intentions for prescribing essential medicines. After analyzing the reliability and validity of the questionnaire, we readjusted the questionnaire statements. Results supported our hypothesis that physicians’ attitudes, PBC and SNs were significant predictors for physicians’ intention to prescribe essential medicines. However, PBC didn’t significantly affect actual behavior to prioritize essential medicines. In general, physicians in sample hospitals showed a passive attitude to providing essential medicines. Through qualitative face-to-face interviews and quantitative analysis, we evaluated 26 observable indicators, and in view of the results we would like to make the following proposals.

There are three kinds of medical insurance in China; namely, urban employee basic medical insurance (UEBMI), urban resident basic medical insurance (URBMI), and the new rural co-operative medical system (NRCMS). Each has its own drug reimbursement list. All essential medicines are included in Class A of the UEBMI list, which means a higher reimbursement of essential medicines than other medicines. The study results indicate that physicians in the county medical institutions preferred to prescribe medicines in the medical insurance catalog. Due to the understanding of medical insurance policy is not clear enough among physicians in county hospitals, and the medical insurance catalog is inconsistent with EM cataloy. Meanwhile, a lack of NEMP comprehension and poor knowledge of essential medicine list (EML) significantly diminished physicians’ intention to select essential medicines as the first choice, they showed less interest for prescribing essential medicine (standardized coefficient was 0.89, 0.72; respectively).

In the present study we identified SNs and PBC as significant and positive predictors of physicians’ intention to prescribe EM. Several important indicators, such as “hospitals promote the EM system (0.83)”, “application of hospital formulary (0.87)”, and “access to EM information (0.82)”, may explain these findings. Effective communication in hospitals plays a major role in physicians’ decision to prescribe EM [25, 26]. It’s critical to establish an effective communication mechanism for NEMP, one that defines coherent criteria for selection of an essential medicine as directors aim to improve quality and safety of drug use. Shanlian Hu [27] suggested the criteria for selecting essential medicines should satisfy the following conditions: designated for prevention and treatment for diseases; quality, safety and clinical efficacy; reasonably priced; convenient to use; and balance between chemical, biological, and traditional Chinese medicines.

Our data show provisions of essential medicines in hospital pharmacies is highly correlated with physicians’ perceived behavior. At present, the bidding prices of essential medicines were lower than actual prices due to the “Two Envelope Selective Tender System” [27]. Physicians didn’t prescribe essential medicines preferentially, the procurement volume was small, tendering used a price–volume agreement system that may persuade the pharmaceutical manufacturers to abandon supplying medicines, thus a shortage of medicines will occur. This phenomenon has a significant influence on the clinical quality and safety, and might deepen physicians’ unsatisfaction towards the essential medicine system. During the structural questionnaire interview, most physicians suggested integrating the drug shortage report into the drug bidding system to incorporate pharmaceutical manufacturers’ credit information system. This would ensure stable provisions of essential medicines in hospital pharmacies.

The effectiveness of the essential medicines’ priority use in the range of public hospitals is ultimately reflected in the behavior of the physicians in prescribing essential medicines. PBC has significantly positive effects on physicians’ intention to prescribe essential medicines. The observable indicator, “recognition of the quality of essential medicine”, indicated part of the reason why essential medicine was considered basic and low-standard product. In China, adjustments to the essential medicine list are made by experts, and the physicians are not knowledgeable on the clinical effects of essential medicines. Consequently, these findings underscore the importance of evidence-based evaluation and dynamic adjustments to the essential medicine list. Drug utilization data and cost-effectiveness analysis based on real world data should be routinely applied in the evaluation of national drug policy (NDP), especially for physicians’ intention for prescription.

Although priority usage of essential medicines is encouraged by the new healthcare reform, it is not mandatory in upper-level hospitals. We used a structural equation to analyze the key factors influencing the priority usage of essential medicines. In order to clarify the main factors affecting the implementation of the essential medicine system in county-level public hospitals at this stage, the repeated variables were deleted, and the key variables were retained during correction model according to the path coefficient. These findings offer insight into rational use of essential medicines comprehensively in public hospitals, and provide a novel methodology for decision-making during the process of public hospital reform.

### Limitation

This study has some limitations. First, although the structural questionnaire interview avoided recall bias and ensured quality of investigation, a small group of respondents showed a lack of interest in the NEMP issue, which resulted in an overall response rate of 80.57% and relatively small sample size. Second, this study focused on only county level hospital, although it reflected the current situation of health care reform, however, this population-based analysis should be conducted in other provinces and the tertiary hospitals to improve the universality of the research results. We are planning more widespread investigations in further studies.

## Conclusion

The structural equation model formed better reflected the main influencing factors of physician prescribing essential medicine. Physician are not aware of the policy design and implementation details on the health care reform. Behavioral attitude influences physician’s intention to prescribing essential medicines significantly but not intensively while subjective norms and perceived behavioral control also influence the prescribing behavior positively.

## Supplementary information

**Additional file 1.** Questionnaire on “Analysis of Factors Affecting Physician’ Priority Prescription of Essential Drugs”.

## Data Availability

The datasets used and/or analyzed during the current study are available from the corresponding author on reasonable request.

## Abbreviations

AB: Attitude towards the behavior
BI: Behavioral intention
CFA: Confirmatory factor analysis
CFI: Comparative-fit index
CIs: Confidence intervals
EM: Essential medicine
EML: Essential Medicine List
GFI: Goodness-of-fit index
IFI: Incremental-fit index
NDP: National Drug Policy
NEMP: National Essential Medicine Policy
NRCMS: the new rural co-operative medical system
PBC: Perceived behavioral control
RAs: Research assistants
RMSEA: Root mean square error of approximation
SEM: Structural equation model
SNs: Subjective norms
TPB: Theory of planned behavior
UEBMI: urban employee basic medical insurance
URBMI: urban resident basic medical insurance
WHO: World Health Organization
