# Supplementary material for: Influencing factors of physicians’ prescription behavior in selecting essential medicines: a cross-sectional survey in Chinese county hospitals
Source: BMC Health Serv Res. 2019 Dec 19;19:980. doi: 10.1186/s12913-019-4831-5 (PMC6923978; doi:10.1186/s12913-019-4831-5)
Supplement: Supplementary file 1 — Additional file 1. Questionnaire on “Analysis of Factors Affecting Physician’ Priority Prescription of Essential Drugs”. [file 12913_2019_4831_MOESM1_ESM.doc]

Additional file 1

Questionnaire on “Analysis of Factors Affecting Physician' Priority Prescription of Essential Drugs”

Hello! Thank you very much for taking the time to fill out this questionnaire! This questionnaire is an ongoing academic study by doctoral students at Anhui Medical University. This study needs to understand your understanding and perception of the factors affecting prescription behavior, and provide a reference for improving the rational use of drugs in medical institutions. Your answer is only for researchers to use in scientific research, answering the right or wrong, please participate and answer!

In addition, this questionnaire is for academic research only and the information is kept confidential. If you would like to know more about the conclusions of this research, please send an email to: [xiexuefeng@ahmu.edu.cn](mailto:xiexuefeng@ahmu.edu.cn) for information. Thank you for your support and help!

Basic Information

JBefore answering, please fill in the following information (please fill in or draw "√" on the serial number):

- Your age: (years)
- Your gender: 🞎 male 🞎 Female
- Your education：¨Specialist ¨Bachelor ¨Master student ¨PhD students and above ¨other
- Your title：¨No title yet ¨Resident ¨Attending physician ¨Deputy Chief Physician ¨Chief physician
- Medical time： year/years
- Average daily outpatient volume： times

The department： ¨Internal medicine ¨surgery 🞎 obstetrics and gynecology ¨pediatrics 🞎 other

Enthusiasm: Each of the following questions (Q) contains several minor questions, and the degree of relevance (or degree of understanding, satisfaction) is divided into three categories (respectively: small, average, large); To quantify, for example: 1 means little impact / dissatisfaction; 2 means influential, but not too big / relatively unsatisfactory; and so on...; 7 means great influence, decisive / very satisfied.

JNote: ‘0’ or ‘√’ can be drawn below the number of influence levels; N/A means the situation is unknown.

**Q1**、The physician’s attitude towards giving priority to prescription essential drugs is affected by the following factors, to what extent?

|  | Small | average | large | N/A |
| --- | --- | --- | --- | --- |
| 01.Your understanding of the essential medicine system | 1 2 | 3 4 5 | 6 7 | 9 |
| 02.Your recognition of the essential medicine system | 1 2 | 3 4 5 | 6 7 | 9 |
| 03.Your satisfaction with the current working status | 1 2 | 3 4 5 | 6 7 | 9 |
| 04.The extent of your actual income change after the medical reform | 1 2 | 3 4 5 | 6 7 | 9 |
| 05.What do you think of the change in income and the elimination of drug addiction? | 1 2 | 3 4 5 | 6 7 | 9 |
| 06.Your satisfaction with changes in income | 1 2 | 3 4 5 | 6 7 | 9 |
| 07.The extent to which different drugs are currently being administered to your income | 1 2 | 3 4 5 | 6 7 | 9 |

Q2、Physicians prioritize the subjective norms of prescription essential drugs, subject to the following factors, to what extent?

|  | Small | average | large | N/A |
| --- | --- | --- | --- | --- |
| 08.Hospital education level for essential drugs | 1 2 | 3 4 5 | 6 7 | 9 |
| 09.The extent of application of the hospital's essential drug list or formulary | 1 2 | 3 4 5 | 6 7 | 9 |
| 10.Hospital incentives for prioritizing essential drugs | 1 2 | 3 4 5 | 6 7 | 9 |
| 11.The role of prescription review system in regulating prescription behavior | 1 2 | 3 4 5 | 6 7 | 9 |
| 12.The proportion of patients who choose their own medicines | 1 2 | 3 4 5 | 6 7 | 9 |
| 13.The extent to which your prescription behavior is affected by patient requirements | 1 2 | 3 4 5 | 6 7 | 9 |
| 14.You and the patient talk about the basic drug situation | 1 2 | 3 4 5 | 6 7 | 9 |

Q3、The extent to which doctors prioritize the control of perceived behavior of essential drugs is affected by the following factors, and to what extent?

|  | Small | average | large | N/A |
| --- | --- | --- | --- | --- |
| 15.Your familiarity with the essential drug list | 1 2 | 3 4 5 | 6 7 | 9 |
| 1. Your access to essential drug information | 1 2 | 3 4 5 | 6 7 | 9 |
| 1. Your understanding of essential medicines | 1 2 | 3 4 5 | 6 7 | 9 |
| 18.Your recognition of the quality of essential medicines | 1 2 | 3 4 5 | 6 7 | 9 |
| 19.Your understanding of the proportion of essential drug reimbursement | 1 2 | 3 4 5 | 6 7 | 9 |
| 1. Hospital pharmacy for the allocation of essential drugs | 1 2 | 3 4 5 | 6 7 | 9 |
| 21.Hospital's supply guarantee for essential drugs | 1 2 | 3 4 5 | 6 7 | 9 |

Q4、The physician’s priority is to prescribe the basic drug’s intentions. What is the following level?

|  | Small | average | large | N/A |
| --- | --- | --- | --- | --- |
| 22.The willingness to participate in basic drug knowledge training | 1 2 | 3 4 5 | 6 7 | 9 |
| 23.What do you think is the importance of learning about essential medicines? | 1 2 | 3 4 5 | 6 7 | 9 |
| 24.Are you willing to provide essential medicines at the time of prescription? | 1 2 | 3 4 5 | 6 7 | 9 |
| 25.Your support for the implementation of the basic drug system in secondary hospitals | 1 2 | 3 4 5 | 6 7 | 9 |
| 26.Your support for zero-rate sales of all drugs in secondary hospitals | 1 2 | 3 4 5 | 6 7 | 9 |

Q5、The physicians’s priority in prescribing the actual behavior of essential drugs is affected by the following factors, to what extent?

|  | Small | average | large | N/A |
| --- | --- | --- | --- | --- |
| 27.The number of basic drug knowledge trainings you have attended | 1 2 | 3 4 5 | 6 7 | 9 |
| 28.The extent to which you actively collect and learn about essential medicines | 1 2 | 3 4 5 | 6 7 | 9 |
| 29.The extent to which you actively recommend essential medicines to patients | 1 2 | 3 4 5 | 6 7 | 9 |
| 30.Priority of choosing essential drugs in actual prescriptions | 1 2 | 3 4 5 | 6 7 | 9 |
| 1. Hospital performance evaluation work | 1 2 | 3 4 5 | 6 7 | 9 |
| 32.Proportion of priority prescription essential drugs in performance appraisal | 1 2 | 3 4 5 | 6 7 | 9 |

Q6、What are your thoughts and valuable suggestions for implementing the basic drug system and the zero rate of drugs in county-level medical institutions?

Thank you for your participation!
